# Risks and familial coaggregation of death by suicide, accidental death and major psychiatric disorders in first-degree relatives of individuals who died by suicide

**DOI:** 10.1192/bjp.2023.85

**Published:** 2023-10

**Authors:** Shih-Jen Tsai, Chih-Ming Cheng, Wen-Han Chang, Ya-Mei Bai, Ju-Wei Hsu, Kai-Lin Huang, Tung-Ping Su, Tzeng-Ji Chen, Mu-Hong Chen

**Affiliations:** Department of Psychiatry, Taipei Veterans General Hospital, Taipei, Taiwan; and Department of Psychiatry, College of Medicine, National Yang Ming Chiao Tung University, Taipei, Taiwan; Department of Psychiatry, Taipei Veterans General Hospital, Taipei, Taiwan; Department of Psychiatry, Taipei Veterans General Hospital, Taipei, Taiwan; Department of Psychiatry, College of Medicine, National Yang Ming Chiao Tung University, Taipei, Taiwan; and Department of Psychiatry, General Cheng Hsin Hospital, Taipei, Taiwan; Department of Family Medicine, Taipei Veterans General Hospital, Taipei, Taiwan; Institute of Hospital and Health Care Administration, National Yang Ming Chiao Tung University, Taipei, Taiwan; and Department of Family Medicine, Taipei Veterans General Hospital, Hsinchu Branch, Hsinchu, Taiwan

**Keywords:** Suicide, familial coaggregation, accidental death, major psychiatric disorders, Taiwan

## Abstract

**Background:**

Evidence suggests a familial coaggregation of major psychiatric disorders, including schizophrenia, bipolar disorder, major depression (MDD), autism spectrum disorder (ASD) and attention-deficit hyperactivity disorder (ADHD). Those disorders are further related to suicide and accidental death. However, whether death by suicide may coaggregate with accidental death and major psychiatric disorders within families remains unclear.

**Aims:**

To clarify the familial coaggregation of deaths by suicide with accidental death and five major psychiatric disorders.

**Method:**

Using a database linked to the entire Taiwanese population, 68 214 first-degree relatives of individuals who died by suicide between 2003 and 2017 and 272 856 age- and gender-matched controls were assessed for the risks of death by suicide, accidental death and major psychiatric disorders.

**Results:**

A Poisson regression model showed that the first-degree relatives of individuals who died by suicide were more likely to die by suicide (relative risk RR = 4.61, 95% CI 4.02–5.29) or accident (RR = 1.62, 95% CI 1.43–1.84) or to be diagnosed with schizophrenia (RR = 1.53, 95% CI 1.40–1.66), bipolar disorder (RR = 1.99, 95% CI 1.83–2.16), MDD (RR = 1.98, 95% CI 1.89–2.08) or ADHD (RR = 1.34, 95% CI 1.24–1.44).

**Conclusions:**

Our findings identified a familial coaggregation of death by suicide with accidental death, schizophrenia, major affective disorders and ADHD. Further studies would be required to elucidate the pathological mechanisms underlying this coaggregation.

Suicide is a major public health and societal problem of global importance.^1,2^ The World Health Organization estimates that 1.2% of all reported deaths in the European Union and 1.4% of global deaths in 2014 were due to suicide.^1^ The global combined years of life lost to suicide were still as high as 30 million years in 2019.^1^ In Taiwan, a country with one of the highest prevalences of suicide mortality (>13/100 000), the prevalence decreased from 19.3/100 000 in 2006 to 15.1/100 000 in 2011 following the implementation of the Taiwan Suicide Prevention Program in 2005.^2^ However, suicide prevalence rebounded and remained at approximately 16/100 000 between 2012 and 2019.^2^

Increasing evidence suggests that suicide and major psychiatric disorders, such as bipolar disorder and major depressive disorder, may aggregate in families. Ballard et al's community-based family study of 1119 adult probands and 5355 first-degree relatives demonstrated that first-degree relatives of individuals with suicide attempts were more likely to be diagnosed with bipolar disorder and major depressive disorder and to attempt suicide, compared with those of the probands who had not attempted suicide.^3^ Mann et al reported that 23.2% of probands with mood disorders who had attempted suicide had a first-degree relative with a history of suicidal behaviour, compared with 13.2% of probands with mood disorders who had not attempted suicide (odds ratio OR = 1.99, 95% CI 1.21–3.26).^4^ However, Joo et al's genome-wide association study of 11 869 individuals of various ethnicities reported that a positive relationship between mood disorders and suicide was present in those of European ancestry but not in those of Asian ancestry.^5^ In addition, Li et al revealed that schizophrenia-related single nucleotide polymorphisms, especially A779C, A218C and A-6526G in the tryptophan hydroxylase gene, may play a crucial role in the pathological mechanism of suicidal behaviours.^6^

Several studies have revealed the common aetiological factors of neurodevelopmental disorders such as attention-deficit hyperactivity disorder (ADHD) and autism spectrum disorder (ASD) and suicidal behaviour.^5,7^ Ljung et al demonstrated that the parents and siblings of individuals with ADHD had increased likelihoods of attempted suicide (parents: OR = 2.42, 95% CI 2.36–2.49; siblings: OR = 2.28, 95% CI 2.17–2.40) and death by suicide (parents: OR = 2.24, 95% CI 2.06–2.43; siblings: OR = 2.23, 95% CI 1.83–2.73) compared with the likelihoods for individuals without ADHD.^7^ Assessing the genome-wide polygenic risk scores for major psychiatric disorders with the suicide risk, Joo et al found a positive association between ASD and suicidal thoughts and behaviours (OR = 1.18, 95% CI 1.06–1.31) among individuals of European ancestry, but not among those of East Asian ancestry.^5^ Whether the familial coaggregation between suicide and major psychiatric disorders, including ADHD, ASD, schizophrenia, bipolar disorder and major depressive disorder, may also be present in the Asian population requires further clarification.

Furthermore, studies have found that individuals with major psychiatric disorders have a higher risk of dying from accidents, such as road traffic accidents, compared with those without psychiatric disorders. A French study by Fond et al of 144 058 trauma-related hospital admissions revealed that patients with schizophrenia and bipolar disorder were at higher risk of 30-day mortality than the control group, probably owing to increased trauma severity.^8^ Sun et al reported that among 391 individuals with schizophrenia who died suddenly or unexpectedly, 12.0% died by accidents and 11.5% died by suicide.^9^ Dalsgaard et al showed that accidents were the most common cause of death among young people with ADHD.^10^ Evidence suggested that people with major psychiatric disorders and their first-degree relatives may share common psychopathological traits, such as impulsivity, inattention and cognitive function deficits, which may imply the possibility of familial coaggregation of major psychiatric disorders, accidental death and suicide. However, whether the risk of accidental death may increase in the first-degree relatives of individuals who died by suicide and whether suicide, accidental death and major psychiatric disorders may cluster in families has never been investigated.

In our study, we linked the Taiwan National Health Insurance Research Database (NHIRD) and the Database of All-cause Mortality for the entire population (*n* = 29 253 529) and investigated the risks of deaths by suicide, accidental death and major psychiatric disorders among the first-degree relatives of individuals who died by suicide. We hypothesised that the first-degree relatives of individuals who died by suicide were more likely to die by suicide or accidents and more likely to be diagnosed with major psychiatric disorders, including ADHD, ASD, schizophrenia, bipolar disorder and major depressive disorder, compared with those having no family history of deaths by suicide.

## Method

### Data source

The Taiwan Health and Welfare Data Science Center of the Ministry of Health and Welfare audited and released the NHIRD, which consists of healthcare data on >99.7% of the entire Taiwan population, for research purposes. The database includes comprehensive information on insured individuals such as demographic data, dates of clinical visits and disease diagnosis. Individual medical records included in the NHIRD are anonymous to protect patient privacy. In the current study, the Longitudinal Health Insurance Database of the NHIRD, which includes all medical records between 2003 and 2017 for the entire Taiwanese population (*n* = 29 253 529), was linked to the Database of All-Cause Mortality, which includes all-cause mortality records between 2003 and 2017 for the entire Taiwanese population for the analyses. Coding in the Database of All-Cause Mortality was as follows: 1, natural death and mortality due to physical diseases (natural cause); 2, accidental death; 3, suicide; 4, homicide; 5, mortality with uncertain cause. Using the method of Cheng et al, family kinships recorded in the NHIRD were used for reconstruction of genealogy.^11^ The diagnostic codes used in the present study are based on the ICD-9-CM (2003–2014) or ICD-10-CM (2015–2017). The Institutional Review Board of Taipei Veterans General Hospital approved the study protocol and waived the requirement for informed consent as this study used deidentified data and no human participant contact was required. NHIRD has been used in numerous epidemiological studies in Taiwan.

### Inclusion criteria and disease classifications

First-degree relatives, including parents (*n* = 15 155), offspring (*n* = 15 013) and siblings (*n* = 38 046), of all individuals who died by suicide between 1 January 2003 and 31 December 2017 were identified as the study group and were assessed for deaths by suicide, accidental death and the following major psychiatric disorders: schizophrenia (ICD-9-CM codes: 295; ICD-10-CM codes: F20, F25), bipolar disorder (ICD-9-CM codes: 296 except 296.2, 296.3, 296.9, and 296.82 or ICD-10-CM codes: F30, F31), major depressive disorder (ICD-9-CM codes: 296.2, 296.3; ICD-10-CM codes: F32, F33), ASD (ICD-9-CM codes: 299.0, 299.8, 299.9; ICD-10-CM codes: F84.0, F84.5, F84.8, F84.9) and ADHD (ICD-9-CM codes: 314; ICD-10-CM codes: F90). These psychiatric disorders were diagnosed at least twice by board-certificated psychiatrists. Suicides and accidental deaths were examined in the Database of All-Cause Mortality. To reduce the confounding effects of age and gender, a 1:4 case–control matched analysis was conducted based on age, gender and familial relationship. For example, an 18-year-old daughter of a mother who died by suicide would be matched with four 18-year-old daughters of mothers without suicide. If this woman had two distinct familial relationships, such as daughter–mother and sister–brother, she would be counted for each of these relationships and matched twice. Income level (levels 1–3 per month: ≤US$1000, US$1001–1800 and ≥US$1801) and urbanisation level of residence (levels 1–4, most to least urbanised) were assessed as a proxy for healthcare availability in Taiwan. Complete NHIRD data on all first-degree relatives of individuals who died by suicide were available from 1 January 2003 or birthdate to 31 December 2017 or death.

### Statistical analysis

For between-group comparisons, the independent *t*-test was used for continuous variables and Pearson's χ^2^ test was used for nominal variables. The prevalence between the two groups was assessed. The relative risks (RR) and 95% confidence intervals (CI) with full adjustment for gender, birth year, income and level of urbanisation were calculated to determine the risks of suicide, accidental death and five major psychiatric disorders between the two groups. Further analyses with additional adjustment for the individual major psychiatric disorders were performed to examine the RRs of suicide and accidental death in the first-degree relatives of individuals who died by suicide compared with the control group. As previously mentioned, each family cluster could contain more than one familial relationship. To manage the effects of clustering, we used a Poisson regression model with a robust error variance to estimate the RRs for clustered data.^12^ Additionally, subanalyses stratified by each kinship (parents, offspring and siblings) were conducted to investigate the risks of mortality and the psychiatric disorders between groups. SAS 9.2 for Windows (SAS Institute, Cary, NC, USA) was used for all statistical analyses, and PROC GENMOD in SAS was used to estimate the adjusted RRs. All tests were two-tailed, and *P* < 0.05 was considered statistically significant.

## Results

In all, 68 214 first-degree relatives of individuals who died by suicide and 272 856 age- and gender-matched controls were included in current study (Table 1). First-degree relatives of individuals who died by suicide had lower income (*P* < 0.001) and resided in less urbanised areas (*P* < 0.001) than the control group (Table 1).

**Table 1:** Demographic characteristics of first-degree relatives of individuals who died by suicide and a matched cohort

	Male			Female			Total		
	FDRs	Matched cohort	*P*	FDRs	Matched cohort	*P*	FDRs	Matched cohort	*P*
Sample, *n*	35 718	142 872		32 496	129 984		68 214	272 856	
Birth year, *n* (%)			>0.999			>0.999			>0.999
Up to 1950	3415 (9.56)	13 662 (9.56)		3454 (10.63)	13 816 (10.63)		6869 (10.07)	27 478 (10.07)	
1951–1960	4524 (12.67)	18 096 (12.67)		3607 (11.10)	14 428 (11.10)		8131 (11.92)	32 524 (11.92)	
1961–1970	4053 (11.35)	16 212 (11.35)		3274 (10.08)	13 096 (10.08)		7327 (10.74)	29 308 (10.74)	
1971–1980	4985 (13.96)	19 940 (13.96)		4471 (13.76)	17 884 (13.76)		9456 (13.86)	37, 24 (13.86)	
1981–1990	8668 (24.26)	34 670 (24.26)		8442 (25.97)	33 768 (25.97)		17 110 (25.08)	68 438 (25.09)	
1991–2000	6608 (18.50)	26 432 (18.50)		6046 (18.61)	24 184 (18.61)		12 654 (18.55)	50 616 (18.55)	
After 2000	3465 (9.70)	13 860 (9.70)		3202 (9.85)	12 808 (9.85)		6667 (9.77)	26 668 (9.77)	
Male, *n*, %	−	−		−	−		35 718 (52.36)	142 872 (52.36)	>0.999
Monthly income, US$: *n*, %			<0.001			<0.001			<0.001
0–1000	14 340 (40.15)	44 529 (31.17)	<0.001	13 296 (40.91)	40 841 (31.42)	<0.001	27 636 (40.51)	85 370 (31.29)	<0.001
1001–1800	13 771 (38.55)	56 256 (39.37)	0.0045	13 092 (40.29)	54 336 (41.80)	<0.001	26 863 (39.38)	110 592 (40.53)	<0.001
≥1801	7607 (21.30)	42 087 (29.46)	<0.001	6108 (18.80)	34 807 (26.78)	<0.001	13 715 (20.11)	76 894 (28.18)	<0.001
Level of urbanisation, *n*, %			<0.001			<0.001			0.0118
1 (most urbanised)	24 199 (67.74)	98 941 (69.26)	<0.001	22 675 (69.78)	92 173 (70.91)	<0.001	46 874 (68.71)	191 114 (70.04)	<0.001
2	8064 (22.58)	31 534 (22.07)	0.0396	6947 (21.38)	27 472 (21.13)	0.3374	15 011 (22.01)	59 006 (21.63)	0.031
3	2306 (6.46)	8419 (5.89)	<0.001	1937 (5.96)	7237 (5.57)	0.0060	4243 (6.22)	15 656 (5.74)	<0.001
4 (most rural)	1149 (3.22)	3978 (2.78)	<0.001	937 (2.88)	3102 (2.39)	<0.001	2086 (3.06)	7080 (2.59)	<0.001

FDR, first-degree relative of an individual who died by suicide.

The Poisson regression model showed that first-degree relatives of individuals who died by suicide were more likely than the matched controls to die by suicide (RR = 4.61, 95% CI 4.02–5.29) and by accident (RR = 1.62, 95% CI 1.43–1.84) and to be diagnosed with schizophrenia (RR = 1.53, 95% CI 1.40–1.66), bipolar disorder (RR = 1.99, 95% CI 1.83–2.16), MDD (RR = 1.98, 95% CI 1.89–2.08) and ADHD (RR = 1.34, 95% CI 1.24– 1.44) (Tables 2 and 3).

**Table 2:** Relative risks of suicide and accidental death for first-degree relatives of individuals who died by suicide and a matched cohort

	Male				Female				All			
	FDRs, *n* (%)	Matched cohort, *n* (%)	ARR (95% CI)	*P*	FDRs, *n* (%)	Matched cohort, *n* (%)	ARR (95% CI)	*P*	FDRs, *n* (%)	Matched cohort, *n* (%)	ARR (95% CI)	*P*
Model 1 Adjusting for demographic data												
Accidental death	264 (0.74)	591 (0.41)	**1.60 (1.38–1.85)**	**<0.001**	86 (0.26)	181 (0.14)	**1.68 (1.30–2.18)**	**<0.001**	350 (0.51)	772 (0.28)	**1.62 (1.43–1.84)**	**<0.001**
Suicide death	300 (0.84)	252 (0.18)	**4.35 (3.67–5.16)**	**<0.001**	178 (0.55)	124 (0.10)	**5.81 (4.62–7.31)**	**<0.001**	478 (0.70)	376 (0.14)	**4.61 (4.02–5.29)**	**<0.001**
Model 2 Additionally adjusting for individual major psychiatric disorders												
Accidental death	264 (0.74)	591 (0.41)	**1.79 (1.55–2.07)**	**<0.001**	86 (0.26)	181 (0.14)	**1.89 (1.46–2.45)**	**<0.001**	350 (0.51)	772 (0.28)	**1.61 (1.42–1.83)**	**<0.001**
Suicide death	300 (0.84)	252 (0.18)	**4.49 (3.78–5.33)**	**<0.001**	178 (0.55)	124 (0.10)	**4.99 (3.94–6.34)**	**<0.001**	478 (0.70)	376 (0.14)	**4.23 (3.68–4.85)**	**<0.001**

FDR, first-degree relative of an individual who died by suicide; ARR, adjusted relative risk. Bold denotes significance at *P* < 0.05.

**Table 3:** Relative risks of major psychiatric disorders for first-degree relatives of individuals who died by suicide and a matched cohort

	Male				Female				All			
	FDRs, *n* (%)	Matched cohort, *n* (%)	ARR^a^ (95% CI)	*P*	FDRs, *n* (%)	Matched cohort, *n* (%)	ARR^a^ (95% CI)	*P*	FDRs, *n* (%)	Matched cohort, *n* (%)	ARR^a^ (95% CI)	*P*
Schizophrenia	396 (1.11)	973 (0.68)	**1.45 (1.28–1.63)**	**<0**.**001**	350 (1.08)	764 (0.59)	**1.63** (**1.43–1.85)**	**<0**.**001**	746 (1.09)	1737 (0.64)	**1.53** (**1.40–1.66)**	**<0**.**001**
Bipolar disorder	387 (1.08)	820 (0.57)	**1.79** (**1.59–2.03)**	**<0**.**001**	522 (1.61)	904 (0.70)	**2.17** (**1.94–2.41)**	**<0**.**001**	909 (1.33)	1724 (0.63)	**1.99** (**1.83–2.16)**	**<0**.**001**
MDD	1014 (2.84)	2153 (1.51)	**1.86** (**1.73–2.01)**	**<0**.**001**	1557 (4.79)	2994 (2.30)	**2.07** (**1.94–2.21)**	**<0**.**001**	2571 (3.77)	5147 (1.89)	**1.98** (**1.89–2.08)**	**<0**.**001**
ASD	128 (0.36)	454 (0.32)	1.18 (0.97–1.44)	0.1023	28 (0.09)	105 (0.08)	1.08 (0.72–1.64)	0.8493	156 (0.23)	559 (0.20)	1.16 (0.97–1.39)	0.0973
ADHD	789 (2.21)	2404 (1.68)	**1.37** (**1.26–1.49)**	**<0**.**001**	209 (0.64)	673 (0.52)	**1.24** (**1.06–1.46)**	**0**.**0077**	998 (1.46)	3077 (1.13)	**1.34** (**1.24–1.44)**	**<0**.**001**

FDR, first-degree relative of an individual who died by suicide; ARR: adjusted relative risk; MDD, major depressive disorder; ASD, autism spectrum disorder; ADHD, attention-deficit hyperactivity disorder. Bold denotes significance at *P* < 0.05.

a. Adjusted for gender, birth year, income and level of urbanisation.

**Fig. 1:**
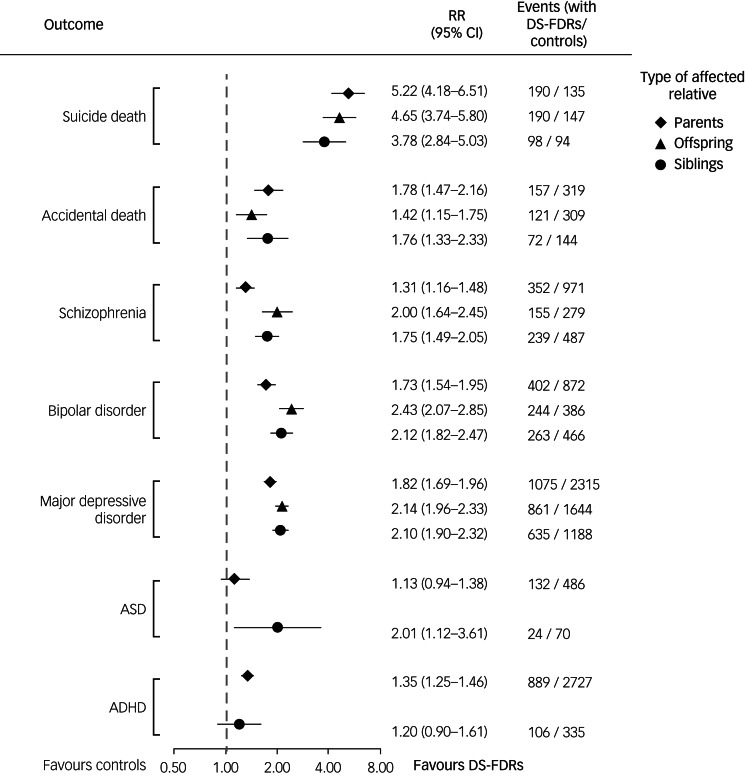
Relative risks of death by suicide, accidental death and major psychiatric disorders for first-degree relatives of individuals who died by suicide and a matched cohort, stratified by kinships. RR, relative risk, adjusted for gender, birth year, income and level of urbanisation; DS-FDR, first-degree relative of an individual who died by suicide; ASD, autism spectrum disorder; ADHD, attention-deficit hyperactivity disorder.

The kinship-stratified analyses showed consistent findings (Fig. 1; Supplementary Tables 1–7, available at https://doi.org/10.1192/bjp.2023.85). Sons of mothers who died by suicide (RR = 10.46, 95% CI 6.08–18.01) and mothers of daughters who died by suicide (RR = 10.33, 95% CI 5.22–20.43) had the highest suicide risk compared with the controls (Supplementary Table 7). Only the siblings of individuals who died by suicide had an increased ASD risk (RR = 2.01, 95% CI 1.12–3.61) compared with the control group (Fig. 1; Supplementary Table 4).

## Discussion

Our findings supported the study hypotheses that the first-degree relatives of individuals who died by suicide were more likely to die by suicide or accidents than the control group. In addition, they had elevated likelihoods of being diagnosed with ASD, ADHD, schizophrenia, bipolar disorder and major depressive disorder compared with the first-degree relatives of those without suicide.

### Psychological, genetic and psychosocial risk factors

Two symptoms present in those who die by suicide, namely cognitive inflexibility (suicidal thoughts) and behavioural impulsivity and disinhibition (suicidal behaviours), may explain the familial coaggregation of suicide with accidental death and major psychiatric disorders. McGirr et al demonstrated that a familial predisposition to suicide was associated with the impulsive–aggressive trait, which also occurs among relatives of individuals who died by suicide, and found that impulsive–aggressive behaviours and suicide attempts aggregated in families.^13^ A small study involving 14 first-degree relatives of people who had died by suicide without a personal history of suicide attempts revealed that they performed poorly in the Wisconsin Card Sorting Test, including more perseverative errors and a lower level of conceptual responses (a decreased responsiveness to changing, yet unambiguous, conditions), compared with those without a family history of suicide.^14^ Hoehne et al reported decision-making impairment in the relatives of people who had died by suicide compared with healthy controls.^15^ Furthermore, Jones et al discovered that adolescents with a family history of suicide attempts had greater deficits in executive function, attention and language reasoning compared with those without such a family history.^16^

Research has identified common genes overlapping with suicide, impulsivity/risk-taking and major psychiatric disorders. A genome-wide association study by Mullins et al of 1683 individuals who had attempted suicide (‘attempters’) and 2946 who had not (‘non-attempters’) with schizophrenia, 3264 attempters and 5500 non-attempters with bipolar disorder and 1622 attempters and 8786 non-attempters with major depressive disorder demonstrated that the polygenic risk score for major depression was associated with suicide attempts in all three disorders.^17^ Koyama et al found that the genetic susceptibility to impulsive aggression was related to suicide ideation regardless of depression comorbidity.^18^ Johnson et al reported that the polygenic risk scores for depression and risk-taking behaviours were significantly associated with suicidal ideation and suicide attempts.^19^ Sokolowski et al further identified that multiple neurodevelopmental genes relating to cell adhesion and migration and receptor tyrosine kinase signalling, such as *BDNF*, *NTRK2*, *CDH9*, *CREB1*, *DLK2* and *NCAM1*, play crucial roles in suicide attempts and major psychiatric disorders.^20^

Finally, suicide, accidental death and major psychiatric disorders may share common psychosocial risks, especially childhood maltreatment and adverse early-life experiences.^21,22^ The stress–diathesis model may explain the biopsychosocial link between psychosocial adversities and the familial coaggregation of suicide, accidental death and major psychiatric disorders.^21^ This diathesis, which exhibits the tendencies to have more suicidal ideation and higher impulsivity, is inherited intergenerationally and evolves into various psychopathologies, such as psychosis, risk-taking, suicidal behaviours and emotion dysregulation, after experiencing childhood maltreatment and other early-life adversities.^21,22^ The stress-resilience model describes a dynamic developmental process encompassing positive adaptation within the context of severe adversity or trauma (e.g. witnessing a suicide), and the model may further explain why so few first-degree relatives of individuals who died by suicide were diagnosed with major psychiatric disorders and died by suicide or accidents in our study.^23^ Interestingly, Orri et al suggested that genetic and unique (non-shared) environmental factors equally contribute to suicidality.^24^

### Limitations

This study had several limitations. First, the incidence of deaths by suicide may be underestimated in the study because the Database of All-Cause Mortality may have recorded several suicides as accidental deaths owing to the lack of definitive suicide evidence. A review based on international data suggested that the three leading cause of death categories that potentially harbour ‘hidden suicides’ are ill-defined or unknown causes of death, events of undetermined intent and accidental deaths.^25^ Second, because information regarding psychosocial stress and environmental factors was unavailable in the database, we could not investigate their effects.

### Implications

Given that our findings suggest familial coaggregation of suicide, accidental death and major psychiatric disorders, clinicians and public health officials should closely monitor the mental health of the first-degree relatives of individuals who died by suicide. Further studies are required to elucidate the pathological mechanisms underlying this coaggregation.

## Data Availability

The data that support the findings of this study are available on request from the corresponding author. The data are not publicly available owing to ethical regulations in Taiwan.

## Supplementary material

For supplementary material accompanying this paper visit https://doi.org/10.1192/bjp.2023.85. click here to view supplementary material
